# Sustainable development an oxymoron? The Political Impact of the Sustainable Development Goals–Transforming Governance Through Global Goals? *by Frank Biermann, Thomas Hickmann, Carole-Anne Sénit*

**DOI:** 10.1007/s10784-023-09598-7

**Published:** 2023-04-04

**Authors:** Joshua Philipp Elsässer

**Affiliations:** Faculty of Economics and Social Sciences, University of Potsdam, Potsdam, Germany

Launched in 2015, the Sustainable Development Goals (SDGs) provide a “shared blueprint” for the world into a sustainable future covering 17 ambitious goals (United Nations, 2022a). These include poverty eradication, zero hunger, good health, quality education, gender equality, access to clean water and energy, decent work and economic growth, sustainable industrialization, reduced inequality, sustainable cities, responsible consumption and production, climate action, life below water and on land, peaceful societies and strong institutions, and finally, cooperation and partnerships for the goals. Succeeding the Millennium Development Goals (MDGs), the SDGs represent a normative, legally non-binding framework, which was agreed by the United Nations (UN) General Assembly for a period of 15 years (ending in 2030). In contrast to the MDGs, the SDGs center on sustainability, highlighting the linkages between the environment with social and economic aspects of development. The broad goals cover most human activities with a total of 169 individual targets. However, many of these targets are qualitative in nature and there is lively debate about goal-specific, universally accepted indicators (or the lack thereof) to measure and assess their progression. Importantly, the SDGs were designed to provide sustainability pathways in an integrated manner across the growing divides with regards to development priorities between the Global South and Global North.

After seven years, how—if at all—have the SDGs worked toward guiding public policy-making and bringing about much needed societal transformations toward a sustainable future for all? Have these ambitious development goals really been as target-oriented and sustainable as their name suggests? In other words, what has been the political impact of the SDGs midterm? With their book “The Political Impact of the Sustainable Development Goals–Transforming Governance Through Global Goals?”, Frank Biermann, Thomas Hickmann, and Carole-Anne Sénit try to provide insights into these questions. Being the first comprehensive study on the SDGs of its kind, the edited volume features contributions from a global coalition of 61 expert scholars and research institutions, such as the dedicated Global Goals Research Project at Utrecht University and the Earth System Governance Network.

In essence, the book is an extensive literature review on studies which capture the political impact of the SDGs across six assessment domains, which also make up the different book chapters: (1) Global Governance, (2) Implementation at Multiple Levels, (3) Interlinkages, Integration and Coherence, (4) Inclusiveness, (5) Planetary Integrity, (6) Indicators & Methods. The author team has screened and evaluated over 3000 scientific studies using the abstract and citation database SCOPUS. Of this initial screening process, a smaller selection of 10 to 20 percent of studies was identified for closer investigation, complemented by an analysis of additional grey literature. Here, the authors do not make clear why and how this literature is consulted. All chapters are similarly structured along three analytical dimensions of steering effects that the authors expect to observe: normative, institutional, and discursive steering effects. Simply put, normative steering effects ask whether there are any observable changes in rules, policies, or programs that are causally linked to the SDGs. Institutional steering effects may be visible through new institutions or arrangements that draw on the SDGs. Discursive steering effects are present if there are changes in the way actors frame or debate sustainability in line with the SDGs.

The overall assessment of the SDG steering effects can be summarized in the following main findings: Thus far, the SDGs have had little transformative impact on policy, rules, programs, or institutions that may spur sustainable development at the international, national, and local level. From a perspective on global governance, the SDGs furthered pre-existing alignment processes within global level institutions (e.g., the UN High Level Political Forum), with many goals building on previous agreements in other policy domains (e.g., within the UN Development System or the UN Environmental Programme). Regarding SDG implementation, the authors notice significant differences across actor groups, geographies, and scales, which lack a coherent pattern of implementation effects, visible for different countries, regions, or levels of governance. These findings demonstrate an apparent mismatch between rhetoric and action: while governments by and large conceive of the SDGs to be highly important, existing development strategies and budgets lack evidence of institutional integration and policy coherence toward the goals. Moreover, the SDGs have not advanced inclusiveness of least developed countries, where vulnerable communities are often discursively prioritized, as exemplified by one of the core SDG values to “leave no one behind” (United Nations, 2022b), but normative effects have yet to catch up with such promises. When it comes to planetary integrity, environmental objectives frequently appear to be deprioritized, reflecting the reality of many growing economies that sacrifice sustainability for development. Such finding uncovers the dominating neoliberal script of sustainable development versus ecological sustainability, where the SDGs have failed to move the needle toward a shared understanding of the centrality of healthy ecosystems as imperative to optimal functioning of social and economic systems.

The authors present more encouraging results on discursive steering effects of the SDGs in a majority of assessment domains. Here, the SDGs have fostered mutual learning among governments and have offered some actors new means to organize, gain support, or mobilize funding—primarily at the local level. In some instances, the SDGs have enabled civil society and non-governmental organizations to hold governments accountable. In sum, such effects are indeed important and may stimulate normative and institutional reform in the future. It may be necessary for public discourse to play out as a precursor to igniting the envisioned transformative potential of the SDGs and meaningful change to occur. However, we do not know whether there is a clear path dependency between discursive effects and the implementation of SDGs within policy, rules, programs, or institutional arrangements (yet)—a question that requires further investigation. Following this hypothesis, the SDG assessment report may be read in a cautiously optimistic way. The authors argue that changing discourses could be seen as a sign of the burgeoning politicization of what sustainable development could entail. Yet, on the contrary, the SGDs might also have depoliticizing effects if the goals are misused by those actors interested in preserving prevalent power asymmetries. In such scenarios, the SDGs might function as a cover-up to paint an overly optimistic picture to the public that these actors are indeed committed to a sustainable and equitable future for all, just so processes of contestation and real transformative change can be kept under control.

All chapters clearly flesh out open questions on the political impact of the goals which should inspire future research on the SDGs. This includes, for instance, more empirical and conceptual work on the drivers and causal pathways of the SDGs to effect change in practice, the influence of non-state actors and partnerships for the SDGs, medium- to long-term impacts of the goals on planetary integrity, or stakeholder-driven analysis on the barriers and effects of entrenched institutional structures that hinder inclusiveness at different scales of governance (as well as a better understanding of how to overcome such barriers). Unresolved questions pertaining to inclusiveness and sustainable development seem to be particularly prevalent and timely. Although the above-mentioned principle of “leaving no one behind” has gained ground in discourse both among policy makers and within civil society, there is little evidence of a transformative impact of the SDGs toward more inclusive and just global institutions that foster the political participation of least developed countries. The authors conclude that “what explains the lack of steering […] on inclusiveness remains unclear” (Sénit et al., 2022, p. 132). Perhaps this finding could at least be partially addressed by widening the focus on studies beyond SCOPUS, a platform that is structurally biased toward research from non-Western countries and non-English language (Tennant, 2020). Given the high profile of the SDG assessment report with a substantial readership from the development studies community, a more dedicated focus with studies on and–importantly–from marginalized research communities of the Global South could complement existing results throughout the book.

Is governance by global goals expedient to stimulate transformative change going forward? Although this question is implicitly referenced by the book’s subtitle, there are no concluding answers. However, the book convincingly demonstrates the need for additional governance tools and mechanisms to make possible societal transformations through SDGs that reconfigure systems across multiple levels and sectors. The loose legal character of the SDGs frequently offers a gateway for actors to interpret the goals in a way that fit their interests, intentionally shaping targets and indicators. There is little evidence of a central authority, such as the UN itself, to steer, harmonize, or call out dysfunctionalities of different interpretations of the SDGs. More effective global goals might thus warrant greater precision and a higher degree of delegation to render institutions more impactful not only at the international level, but also through dedicated ministries and cross-sectoral coordination at the national level. At the same time, such rather focused approaches need to be complemented by more meaningful integration across a variety of institutions at the international level. For instance, in the area of global environmental governance, the growing siloization of specialized multilateral environmental agreements has fostered a culture of “either, or”. However, pathways toward global sustainability will necessitate increased cooperation across issue areas to fill governance gaps and harness synergies given the interdependencies between environmental problems. This could entail a stronger coordination across UN agencies and/or support from non-state actors as well as transnational networks and initiatives.

From a more pessimistic perspective, recent global challenges, such as the COVID-19 pandemic or the Russo-Ukrainian War, suggest that different development priorities may ultimately overshadow meaningful change at both policy and institutional dimensions. Cynics might argue that humanity will never reach sustainable development as laid out under the SDGs given the experience of a slow-paced, incremental process in some policy areas that deal with transboundary environmental problems over the past decades. For example, intergovernmental climate change negotiations are dragged by competing interests where climate change is juxtaposed with other human development priorities. This includes dealing with poverty eradication and hunger in least developed nations at one extreme, and ever-rising (albeit at a somewhat slower rate) production and consumption levels to counter a looming economic recession feared by some major carbon-emitting nations at the other. In global climate negotiations, economic interests have most frequently trumped social and environmental concerns. In effect, climate change appears to be a wicked problem to solve, but it is dwarfed by the myriad complexities of what sustainable societal transformations necessitate. Therein lies the challenge for sustainable development: Since the first Club of Rome Report in 1972, we know that depleting natural resources and destructed ecosystems ultimately set limits to development and growth (Meadows et al., 1972). Whereas growth—and this will entail carbon-intensive practices to some extent—is necessary for developing nations, developed countries need to drastically scale back their expansionist behavior and reduce unsustainable practices. Taking these great challenges and thus far rather modest political impacts of the SDGs into account, “The Political Impact of the Sustainable Development Goals–Transforming Governance Through Global Goals?” raises the question: is sustainable development an oxymoron?
